# Peripheral Upregulation of Parkinson’s Disease-Associated Genes Encoding α-Synuclein, β-Glucocerebrosidase, and Ceramide Glucosyltransferase in Major Depression

**DOI:** 10.3390/ijms25063219

**Published:** 2024-03-12

**Authors:** Razvan-Marius Brazdis, Claudia von Zimmermann, Bernd Lenz, Johannes Kornhuber, Christiane Mühle

**Affiliations:** 1 Department of Psychiatry and Psychotherapy, Universitätsklinikum Erlangen and Friedrich-Alexander University Erlangen-Nürnberg (FAU), 91054 Erlangen, Germany; razvanmarius.brazdis@uk-erlangen.de (R.-M.B.); bernd.lenz@zi-mannheim.de (B.L.); johannes.kornhuber@uk-erlangen.de (J.K.); 2 Department of Addictive Behavior and Addiction Medicine, Central Institute of Mental Health (CIMH), Medical Faculty Mannheim, Heidelberg University, 68159 Mannheim, Germany

**Keywords:** affective disorder, anxiety, biomarker, *GBA1*, gene expression, major depressive disorder, MDD, PD, *SNCA*, *UGCG*

## Abstract

Due to the high comorbidity of Parkinson’s disease (PD) with major depressive disorder (MDD) and the involvement of sphingolipids in both conditions, we investigated the peripheral expression levels of three primarily PD-associated genes: α-synuclein (*SNCA*), lysosomal enzyme β-glucocerebrosidase (*GBA1*), and UDP-glucose ceramide glucosyltransferase (*UGCG*) in a sex-balanced MDD cohort. Normalized gene expression was determined by quantitative PCR in patients suffering from MDD (unmedicated *n* = 63, medicated *n* = 66) and controls (remitted MDD *n* = 39, healthy subjects *n* = 61). We observed that expression levels of *SNCA* (*p* = 0.036), *GBA1* (*p* = 0.014), and *UGCG* (*p* = 0.0002) were higher in currently depressed patients compared to controls and remitted patients, and expression of *GBA1* and *UGCG* decreased in medicated patients during three weeks of therapy. Additionally, in subgroups, expression was positively correlated with the severity of depression and anxiety. Furthermore, we identified correlations between the gene expression levels and PD-related laboratory parameters. Our findings suggest that *SNCA*, *GBA1*, and *UGCG* analysis could be instrumental in the search for biomarkers of MDD and in understanding the overlapping pathological mechanisms underlying neuro-psychiatric diseases.

## 1. Introduction

Major depressive disorder (MDD) is one of the most severe and common psychiatric disorders. It strongly impairs psychosocial functioning and diminishes quality of life. Depression has an extreme global economic burden and has been listed as one of the leading causes of disability by the World Health Organization (WHO). According to the WHO, by 2030, depression will be the leading cause of disease burden in high-income countries [1]. A major depressive episode (MDE) is characterized by a combination of affective, social, and somatic symptoms, such as persistently low or depressed mood, anhedonia or decreased interest in pleasurable activities, fatigue, feelings of guilt or worthlessness, poor concentration, changes in appetite and weight, sleep disturbances, and/or suicidal thoughts [2]. A variety of pathomechanisms have been proposed to underlie the etiology of the disease and the effects of antidepressants. These mechanisms span from disrupted monoamines, oxidative pathways, and the hypothalamic–pituitary–adrenal axis to neurotrophic homeostasis and inflammatory processes [3,4]. Additionally, prenatal factors are believed to augment the risk for depression and suicide in adulthood [5]. In recent years, considerable evidence has emerged regarding the dysregulation of sphingolipid metabolism in depression and comorbid anxiety [6,7], supported by findings from animal models [8,9,10] and clinical studies revealing significantly elevated levels of the central sphingolipid ceramide in patients with MDD, alongside its correlation with the severity of depression [11,12].

Depression is also one of the most important and frequent non-motor symptoms in Parkinson’s disease (PD) with an overall prevalence of about 40% [13,14]. PD is the most common neurodegenerative movement disorder affecting 1–2% of the population aged over 65 years [15]. Age is considered to be the greatest risk factor for PD [16] and according to sociodemographic developments, the incidence rate of PD increases. Over the course of their PD, male patients experience neuropsychiatric disturbances, including depression, anxiety, sleep disturbances, psychosis, and behavioral and cognitive changes [17]. The main neuropathological hallmark of PD is the presence of eosinophilic neuronal inclusions, mainly consisting of abnormal aggregated α-synuclein (α-Syn) and known as Lewy bodies (LBs) [18]. α-Syn is a small (~14 kDa) presynaptic soluble neuronal protein that aggregates through monomers, oligomeric intermediates to insoluble amyloid fibrils and finally deposits along with other proteins into LBs [19]. α-Syn gene (*SNCA*) missense mutations, as well as its duplication and triplication, cause early-onset parkinsonism [20], pointing out the importance of α-Syn pathology for PD and other synucleinopathies.

PD is also intricately connected to sphingolipid metabolism. Abundantly present in the eukaryotic cell membrane, sphingolipids also play a crucial role in diverse cellular processes, including cell differentiation, signal transduction, and apoptosis [21]. Reduced activity of acid sphingomyelinase, which catalyzes the hydrolysis of sphingomyelin to ceramide, has been linked to an earlier onset of PD [22]. Different mutations of the corresponding gene *SMPD1* have been associated with an increased risk for PD in the Ashkenazi Jewish as well as Chinese populations [23]. Mutations in the β-glucocerebrosidase gene (*GBA1*) that encodes for the lysosomal enzyme β-glucocerebrosidase (GCase) are considered to be the most important genetic risk factor for PD [24]. Functional loss of GCase, which cleaves glycosylceramide to yield ceramide, has been linked with increased α-Syn aggregation and neurotoxicity [25]. Accordingly, pharmacologically driven reduction in glycosphingolipid levels in a human-induced pluripotent stem cell-derived model can revert toxic α-Syn oligomers to their normal physiological forms [26]. Moreover, UDP-glucose ceramide glucosyltransferase (*UGCG*), the enzyme responsible for the de novo synthesis of glucosylceramide, is also associated with several diseases including PD [27]. Mechanistically, sphingolipids are thought to act as modulators of α-Syn aggregation as they could compete with α-Syn for binding sites on cellular membranes, potentially altering membrane fluidity and protein trafficking, or directly with α-Syn, modulating its folding and aggregation [28]. For example, the GCase substrate glycosylceramide was found to directly stabilize the soluble oligomeric intermediate form of α-Syn. Vice versa, α-Syn inhibited the activity of wild-type GCase such that the bidirectional effect may lead to a self-propagating disease [29,30].

Another hallmark of PD is the progressive degeneration of dopaminergic neurons in the substantia nigra pars compacta [31]. Dopamine is critical for movement, and its progressive loss causes the cardinal motor features of PD, including bradykinesia, rigidity, tremor, and postural instability [32]. The non-motor symptoms such as depression, anxiety, or psychosis are based on the dysregulation of dopaminergic processes of mainly mesocortical and mesolimbic pathways, non-dopaminergic processes, and their interactions [33,34]. The subsequent risk of PD is significantly higher in patients with depression as compared with healthy individuals [35,36]. These connections may run deeper, as PD may directly precipitate mood disorders. Alterations in the brain due to PD can trigger depression and anxiety. Also, depression can exacerbate other PD symptoms. Notably, approximately 12–37% of PD patients receive a depression diagnosis before the clinical identification of their motor symptoms [37].

The high incidence of comorbidity suggests that depression and PD may be related by a shared cellular pathway involving sphingolipids and α-Syn. Despite accumulating evidence supporting the involvement of sphingolipids in PD [38,39], research examining PD-related genes in MDD remains limited. This prompted us to conduct an analysis of gene expression, with a special focus on *SNCA*, *GBA1*, and *UGCG*. We utilized samples from our large, sex-balanced cohort of patients experiencing an MDE, along with matched controls, to investigate their potential as biomarkers for depression and anxiety. Building on previous research, our main hypothesis posited group differences in the expression of these three genes within peripheral blood cells between depressed patients and healthy individuals. Upon observing these differences, we delved deeper into the associations of gene expressions with the severity of depression and anxiety. Additionally, we studied their correlations with laboratory parameters related to PD.

## 2. Results

### 2.1. Cohort Characteristics

The study involved a cohort of 230 patients and controls with 227 individuals providing at least one gene expression data point for analysis (see Table 1). Sex distribution was evenly balanced among unmedicated and medicated patients as well as healthy controls, except for remitted patients with substantially more females than males. Overall, there were no significant differences in age, education years, or body mass index (BMI) between currently depressed patients and the combined group of remitted patients and healthy controls. However, medicated patients presented with higher BMI, likely due to antidepressant side effects. Elevated scores across all three depression scales (Hamilton Depression Rating Scale (HAM-D), Montgomery and Åsberg Depression Rating Scale (MADRS), and Beck Depression Inventory-II (BDI-II)) highlighted the medium to high severity of depression among patients with a current MDE. These scores showed a decline from baseline to follow-up approximately 3 weeks later (all *p* < 10^−7^), indicating therapeutic effectiveness. Remitted patients displayed lower depression scores compared to those with current MDE, although scores remained higher than those observed in healthy controls.

There were no significant differences in the gene expression of *SNCA*, *GBA*, and *UGCG* between males and females, neither in the entire cohort nor within subgroup analyses (all *p* > 0.088, Table 1), enabling a combined assessment. Moreover, none of the gene expressions at T1 was associated with age, duration of education, or body mass index in any of the four groups (all *p* > 0.074).

### 2.2. Elevated Gene Expression of SNCA, GBA1, and UGCG in Depressed Patients

**Figure 1:**
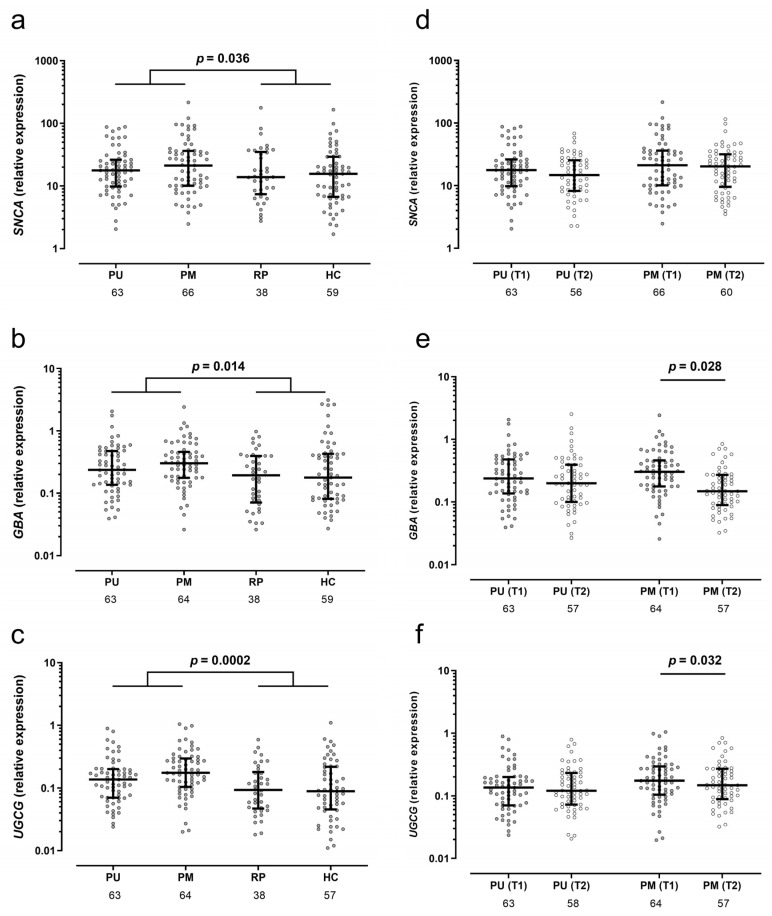
Peripheral gene expressions of *SNCA* (**a**), *GBA* (**b**), and *UGCG* (**c**) were significantly higher in patients with current MDE (combined unmedicated patients (PU) and medicated patients (PM)) at inclusion compared to unaffected individuals (combined remitted patients (PR) and healthy subjects (HC)). These levels remained for *SNCA* (**d**) but decreased between inclusion (T1) and follow-up (T2) after on average three weeks of treatment as usual for *GBA1* (**e**) and *UGCG* (**f**) in the group of initially medicated patients. Normalized gene expression relative to reference genes is shown on a logarithmic *y*-axis. The numbers of individuals are provided below the *x*-axis. *p*-values from Mann–Whitney U test (**a**–**c**) and Wilcoxon test for paired values (**d**–**f**). Box plots with median and interquartile range.

In the absence of sex-based differences, our initial analysis compared all patients experiencing a current MDE (unmedicated and medicated) with controls devoid of depression (patients with remitted MDD and healthy controls). We identified significantly elevated expression levels of all three genes in individuals with depression: *SNCA* (+27%, *p* = 0.036), *GBA1* (+56%, *p* = 0.014), and *UGCG* (+75%, *p* = 0.0002) (Figure 1a–c). In exploratory analyses stratified by sex, we noted similarly heightened levels in both male and female subgroups, with *UGCG* exhibiting the most pronounced effects. Upon subgroup comparison, healthy controls and remitted patients did not display significant differences; however, medicated patients exhibited slightly elevated expression levels (20–29%) compared to unmedicated patients, with statistical significance observed solely for UGCG (*p* = 0.028, Supplementary Table S1).

Throughout the three-week period of standard treatment until the follow-up assessment, elevated levels did not revert to baseline in patients (Figure 1d–f). Notably, a significant reduction in levels was observed solely among medicated patients (both males and females), with *GBA* decreasing by 21% (*p* = 0.028) and *UGCG* by 23% (*p* = 0.032) for the total groups.

### 2.3. Associations of SNCA, GBA1, and UGCG Expressions with Depression Severity and Anxiety

Based on the elevated gene expression levels observed in depressed patients compared to unaffected individuals, we conducted an exploratory analysis to determine whether high gene expression levels correlated with depression severity as assessed by clinicians (HAM-D, MADRS) or self-evaluation (BDI-II) with subsequent sex-stratified analysis in the event of nominally significant effects. However, we found that only *SNCA* mRNA levels exhibited a significant positive correlation with BDI-II scores, but not with other scales, in the group of medicated and unmedicated patients at inclusion (ρ = 0.190, *p* = 0.031) and in the female subgroup (ρ = 0.256, *p* = 0.034, see Table 2). Furthermore, we did not observe any significant predictive association between gene expression levels and follow-up scores or their changes during therapy or associations with anxiety in patients with MDE.

**Figure 2:**
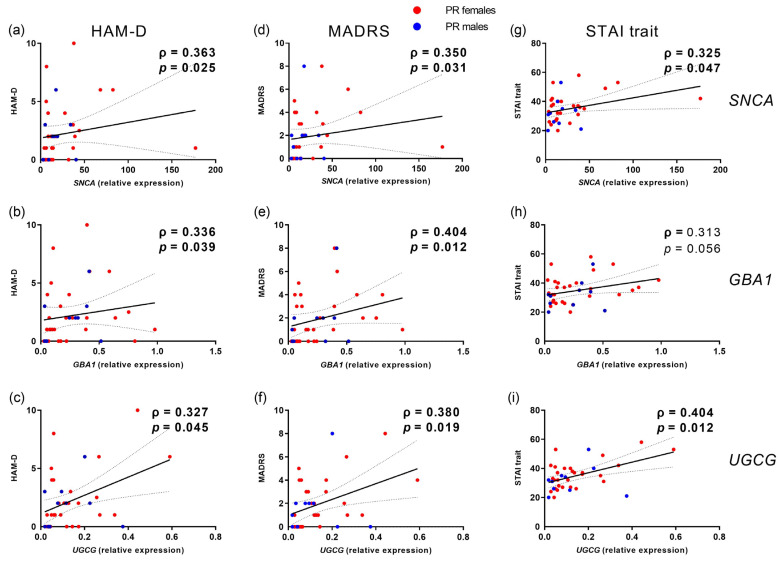
Positive correlations between depression severity assessed by HAM-D (**a**–**c**), MADRS (**d**–**f**), and STAI trait (**g**–**i**) with peripheral gene expressions of *SNCA*, *GBA*, and *UGCG* in patients with remitted major depressive disorder (PR) separated in female (red dots) and male (blue dots) subgroups at inclusion. Linear regression line for the combined group with 95% confidence interval and statistics (Spearman correlation, in bold for *p* < 0.05). Sex-stratified statistical data are in Table 3.

Notably, elevated expression levels of *SNCA*, *GBA1*, and *UGCG* genes correlated with higher HAM-D and MADRS depression scores in patients with remitted MDD, observed across the entire cohort and specifically in the female subgroup (refer to Table 3). These associations demonstrated consistently moderate effects (Figure 2).

Consistent with the high prevalence of comorbidity between depression and anxiety, sum scores for trait anxiety (with a theoretical range of 20–80) were elevated, nearly doubling in both medicated and unmedicated patients compared to healthy controls (Table 1), surpassing the suggested cut-off point of 39–40 for clinical significance [40]. Therefore, akin to depression severity scores, we examined the correlation between *SNCA*, *GBA1*, and *UGCG* expressions and anxiety scores. Remarkably, only among remitted patients, elevated *SNCA* and *UGCG* expression levels were associated with higher trait anxiety scores at baseline (see Table 3). This association was observed specifically in female patients, with no significant correlation noted in males (Figure 2).

### 2.4. Associations of SNCA, GBA1, and UGCG Expressions with Routine Blood Parameters

In our exploratory analysis, we investigated potential correlations between PD-related gene expression levels and routine blood parameters, particularly those with a potential link to PD. We identified a weak trend associating *SNCA* and *GBA1* expressions with human serum albumin levels in depressive patients at inclusion (ρ = 0.164, *p* = 0.064, and ρ = 0.157, *p* = 0.079, respectively), which appeared stronger in the male subgroup (ρ = 0.315, *p* = 0.014; ρ = 0.230, *p* = 0.077), and particularly evident in medicated patients (ρ = 0.287, *p* = 0.019; ρ = 0.255, *p* = 0.042).

In relation to blood calcium levels, we observed sex-specific effects, with contrasting correlations noted for male (ρ = −0.548, *p* = 0.006) and female (ρ = 0.336, *p* = 0.065) currently depressed patients who were unmedicated at inclusion, concerning the relative change in *SNCA* expression levels from inclusion to follow-up.

Additionally, we uncovered a robust correlation between gene expression and serum lactate dehydrogenase activity, particularly notable among males within the subgroup of patients with remitted MDD (refer to Table 4).

Lastly, the expression levels of the three genes exhibited associations with creatine kinase in individuals currently not affected by depression (see Table 4), displaying a notably stronger negative correlation among male healthy controls for *GBA1* and *UGCG* (ρ = −0.410, *p* = 0.024 and ρ = −0.427, ρ = 0.023, respectively).

## 3. Discussion

In this study, we investigated the expression of the PD-related genes *SNCA*, *GBA1,* and *UGCG* in human blood cells of MDD patients and matched controls and their association with the severity of depression, anxiety, and PD-related laboratory parameters. Our principal finding reveals significantly elevated expression levels of all three genes among moderately to severely depressed patients (*n* = 129) compared to a combined group consisting of patients with remitted MDD and healthy controls (*n* = 97). The increased levels of *SNCA* mRNA are in line with a previous smaller study involving 70 patients and 18 controls [41]. This is also supported by the detection of higher α-Syn protein levels in serum samples of 132 inpatients with MDD compared to controls independent of age suggesting that depression could influence α-Syn metabolism and thus possibly not only function as a prodromal symptom of Lewy body dementia but also as a potential causal risk factor [42]. Furthermore, the involvement of *SNCA* in psychiatric disorders was also observed in a study of eating disorders, which demonstrated a positive correlation between the severity of depressive symptoms and *SNCA* mRNA levels [43].

A potential mechanistic explanation for the linkage of *SNCA* to MDD involves the negative modulation of serotonin transporter activity by α-Syn through the formation of heteromeric complexes via direct protein–protein interactions, as suggested by co-immunoprecipitation studies, and the subsequent reduction in transporter levels at the plasma membrane due to increased *SNCA* expression [44]. Additionally, α-Syn has been observed to influence the activity and trafficking of the norepinephrine transporter, a process contingent upon its interactions with microtubules [45]. The translation of elevated *SNCA* expression levels into modifications of α-syn conformational states and bioavailability, as well as the correlation between peripheral and central protein levels, necessitate further investigation. Given that studies in rats exhibiting depressive-like symptoms have revealed an upregulation of γ-Syn in the frontal cortex [46], exploration of additional members of the synuclein family, namely β- and γ-synuclein, in patients is warranted.

To our knowledge, this study represents the first demonstration of elevated *GBA1* (*p* = 0.014) and *UGCG* (*p* = 0.0002) mRNA expression levels in patients experiencing a current MDE compared to patients with remitted MDD and healthy controls. Importantly, these elevations persist even after conservative correction for multiple tests using the Bonferroni method, thus reinforcing the robustness of our findings. While the observed decrease in both *GBA1* and *UCGC* expression levels during the three-week course of antidepressive therapy, leading toward normalization, was only noted in the subgroup of patients already receiving medication at the time of inclusion, it further underscores the potential involvement of these PD-related sphingolipid enzymes in MDD. It is worth noting that the relatively short time frame of three weeks may have limited the magnitude of effects, particularly in the group of patients who were not receiving medication at the time of inclusion. While data on *UGCG* in PD is limited, GCase (*GBA1*) has been thoroughly investigated, with lower activities associated with increased aggregation of α-Syn [25] and thus pathological conditions. Accordingly, overexpression of GCase in the central nervous system in mice reduces α-Syn levels with the potential to modulate the progression of alpha-synucleinopathies [47]. In this context, discovering elevated levels of *GBA1* expression in individuals with the disease may seem unexpected and potentially protective for MDD patients and/or could also be part of a negative feedback loop regulating actual protein levels of the enzyme. Most studies have primarily focused on reduced GCase activity as it is strongly linked to PD. However, a study utilizing the small molecule chaperone ambroxol has demonstrated an increase in GCase activity, which corresponded with approximately a 20% decrease in both α-Syn and phosphorylated α-Syn protein levels in mice [48]. It is intriguing to observe an elevation in the expression levels of enzymes catalyzing opposing reactions, such as the de novo synthesis of glucosylceramide (*UGCG*) versus its cleavage (*GBA1*). However, such a phenomenon could be interpreted as indicative of increased turnover and/or the possibility that the specific localization of these sphingolipids may elicit distinct effects.

The potential involvement of α-Syn in MDD is further substantiated by the positive correlation observed between depression severity (self-assessment by BDI-II) and *SNCA* expression levels in both medicated and unmedicated patients. Even in patients with remitted MDD, where depression severity, as assessed by clinicians using HAM-D and MADRS, is notably lower, expression levels of all three genes still exhibit positive associations with medium effect sizes ranging from 0.3 to 0.4 (Spearman’s ρ).

Prior investigations indicate a notable comorbidity between depression and anxiety, with rates reaching up to 50–80% [49,50]. Strong evidence supports the existence of similar pathological mechanisms underlying both depressive and anxious disorders. They share numerous genetic [51], familial, and environmental risk factors [52], and are frequent features observed in PD. While anxiety risk in PD has been less extensively studied compared to depression, numerous studies highlight its high prevalence [53]. In our assessment, we evaluated trait anxiety using the STAI and noted that elevated *SNCA* and *UGCG* expressions coincided with high anxiety scores specifically within the group of remitted patients—similar to the associations identified for depression severity scores, with comparable effect sizes. This link was observed only for trait anxiety, a more stable personality characteristic, as opposed to state anxiety, which represents a temporary feeling and exhibits weaker or absent correlations with these gene expressions.

In an exploratory approach, we examined correlations between gene expression levels and laboratory parameters related to PD. Human serum albumin, known to decelerate α-Syn aggregation [54], was also identified within a 2D gel protein biomarker panel designed to differentiate PD patients from age-matched controls based on blood serum proteins [55]. In our study, we noted a positive correlation between serum albumin levels and *SNCA* expression among patients, particularly within the subgroup of male patients receiving medication.

Neuronal calcium homeostasis plays a pivotal role in regulating aging, and neurodegeneration, and also in triggering α-Syn oligomerization [56]. While numerous studies link elevated neuronal calcium levels with α-Syn aggregation, their peripheral interaction remains relatively understudied and less understood. Some investigations have highlighted a correlation between hypocalcemia and the severity of PD [57]. In our study, we identified a robust inverse correlation between the relative expression change in *SNCA* during therapy and serum calcium values in both male and female patients. Although this represents the sole contrasting sex-specific effect observed in our investigation, it is important to note that different biological mechanisms in males and females regarding depression have been documented [58] and warrant further exploration. Moreover, the concept of masculine depression, which refers to alternative depression symptoms more commonly associated with the male gender, such as externalizing behaviors, emotional suppression, substance misuse, and risk-seeking [59,60,61], deserves further attention.

Elevated lactate dehydrogenase (LDH) activity has been associated with neurodegenerative disorders, including PD, potentially reflecting neuronal damage and inflammation [62]. On the other hand, reduced levels of serum LDH were found to be associated with depression and suicide attempts [63]. While a definitive connection between *SNCA*, *GBA1*, *UGCG* expression, and LDH has not yet been clearly established [64,65], we observed a robust correlation between the expression of these genes and serum LDH levels in our male patients with remitted MDD.

Creatine kinase (CK) is one of the primary kinases involved in promoting α-Syn phosphorylation [66]. Studies have indicated that PD patients exhibit elevated levels of serum CK [67]. Moreover, the serum ubiquitous but not the sarcomeric forms of the mitochondrial CK activity were significantly decreased in PD patients compared to controls and correlated significantly with the disease progression rate, duration, and age at onset [68]. Interestingly, in our male control group, we observed a strong negative correlation between the expression levels of *SNCA*, *GBA1*, *UGCG*, and CK.

Our study exhibits several strengths. Firstly, the size of the sex-balanced cohort, encompassing the four subgroups and the monitoring of the treatment course, has to be highlighted. Although the follow-up period of approximately 3 weeks was relatively short and a longer duration would aid in the potential identification of (stronger) effects, the second visit still enabled us to analyze the predictive potential of gene expression. Another strength lies in the inclusion of three scales for depression severity, either rated by a clinician or through self-evaluation. Moreover, we meticulously excluded psychiatric comorbidities such as post-traumatic stress disorder, along with patients taking anti-inflammatory medications. These measures contribute to the robustness and specificity of our findings.

The present study is subject to several limitations. While the primary and novel finding of increased gene expression levels for *GBA* and *UGCG* in depressed patients remained significant after correction for multiple testing, the majority of our observations remain at an exploratory level with small to medium effect sizes. Additionally, the associational study design restricts our ability to draw causative conclusions or assume direct physiological links with gene expressions. Further investigations could include the analysis of protein levels of α-Syn in these samples [69,70,71] or isolated exosomes [72], as well as activities of the respective enzymes and their associations with sphingolipid levels of their substrates and products. It could be informative to extend activity assays to further enzymes of the sphingolipid pathway such as acid and neutral ceramidases and sphingomyelinases. Recent evidence suggests the latter to be detectable in human serum/plasma samples [73]. Neutral sphingomyelinase was also found to be strongly reduced in the hippocampus of PD-induced mice in association with neuroinflammation [74]. Moreover, inhibition of acid ceramidase in GC-ase deficient patient-derived dopaminergic neurons resulted in increased ceramide, and decreased glycosylsphingosine levels eventually reduced oxidized α-Syn levels suggesting acid ceramidase inhibition as a potential therapeutic strategy for synucleinopathies linked to *GBA1* mutations [75]. In addition, exploring the pattern of splice variants and their effects could be valuable areas for future research. Moreover, we cannot exclude the possibility that observed alterations represent adverse metabolic effects of psychotropic drugs. Therefore, investigating the effect of nonpharmacological antidepressant treatments such as music therapy (Behzad et al., manuscript submitted) or bouldering and mental models [76] on the expression of PD-related genes could provide valuable insights. Given that up to 30% of patients with major depressive disorder are therapy refractory, which could be also related to inflammatory processes [77], it is crucial to further explore the underlying pathological mechanisms and their interactions with immune responses and stress, which are important factors in major depressive disorder and PD pathologies [78]. Additionally, our cohort, recruited at a university hospital from an academic surrounding, might not be fully representative of patients and healthy controls in other regions or cultures. Replication and extension of these results are certainly warranted in patients of both sexes, with sufficient group size to differentiate between different therapeutic approaches. We also acknowledge that the analysis of expression levels in peripheral leukocytes may not fully reflect the pathophysiological processes occurring within the central nervous system. Mouse models of PD and MDD, with easier access to region-specific brain material, could further substantiate the investigation of the interaction between sphingolipid and α-Syn pathology [79].

## 4. Materials and Methods

### 4.1. Study Description

The study was conducted utilizing samples and data from the CeraBiDe (“Ceramide-associated Biomarkers in Depression”) study [80,81,82,83,84]. Recruitment took place between 01/2014 and 01/2017, adhering to the ethical principles outlined in the sixth revision of the Declaration of Helsinki (Seoul 2008) and the International Conference on Harmonization Guidelines for Good Clinical Practice (1996) and had received approval from the Ethics Committee of the Medical Faculty of the Friedrich-Alexander University Erlangen-Nürnberg (FAU, ID 148_13 B, 17 July 2013). Written informed consent was obtained from all participants. Depressed patients were recruited from both inpatient and outpatient facilities of the Department of Psychiatry and Psychotherapy at the Universitätsklinikum Erlangen, along with other individuals meeting the inclusion criteria. Healthy control subjects were recruited from the local community through various means of advertisement and underwent a rigorous screening process to exclude severe somatic and psychiatric morbidity, except nicotine dependence. The study included 130 patients with a current MDE, of which 64 were not taking any antidepressants for at least 2 weeks, while 66 were on stable antidepressant regimes for the same duration. Additionally, 61 healthy control subjects and 39 patients with remitted MDD were included in the study. Remitted MDD patients were individuals with a first MDE onset before the age of 60 and no depressive episode in the preceding 12 months. Inclusion criteria comprised age between 18 and 75 years and a BMI of 18.5–35.0 kg/m^2^, while exclusion criteria included severe physical illness, autoimmune disorders, pregnancy, breastfeeding, and recent use of anti-inflammatory drugs or corticosteroids within the last 7 days (details provided in [17]). All participants underwent screening using the structured clinical interview for DSM-IV (SKID-I), blood sampling, and psychometric tests at inclusion (T1). A subset of 59 unmedicated and 60 medicated patients participated in a direct follow-up with blood sampling and psychometric scales 21 and 19 days post-inclusion (median), with interquartile ranges of 17–28 and 15–24, respectively (T2). Throughout the observation period, all patients received treatment as usual, including adjustments to psychotropic drug administration for some individuals (details provided in [81]).

### 4.2. Psychometric Scales

The depression severity was measured by trained staff using the 17-item version of Hamilton Depression Rating Scale (HAM-D, [85]) and the 10-item Montgomery and Åsberg Depression Rating Scale (MADRS, [86]). In addition, it was self-reported with the Beck Depression Inventory-II (BDI-II, [87]). The levels of trait and state anxiety were self-evaluated by employing the 40-item State–Trait Anxiety Inventory (STAI) [88].

### 4.3. Collection and Analysis of Blood Samples

Blood samples were obtained from all individuals in the morning following an overnight fast to reduce circadian variations at inclusion (T1) and at follow-up (T2). PAXgene TM Blood RNA tubes (Qiagen, Hilden, Germany) were stored at −80 °C for later isolation of RNA. Separate vials were collected for routine laboratory tests, which were carried out at the accredited Central Laboratory of the University Hospital Erlangen, Germany, in accordance with DIN EN ISO 15189 standards [89].

### 4.4. Gene Expression Analysis by Quantitative PCR

The PAXgene TM Blood RNA Kit (Qiagen, Hilden, Germany) was used to isolate total RNA from PAX blood tubes according to the manufacturer’s instructions. The concentration and purification of RNA were determined photometrically using a NanoDrop ND-1000 UV–Vis spectrophotometer (Peqlab, Erlangen, Germany), and the integrity of representative samples was verified on agarose gels in adherence with accepted guidelines for quality control [90]. Five hundred nanograms of RNA were used in a 20 µL reverse transcription reaction using the Quanta cDNA Kit (Gaithersburg, MD, USA) to synthesize cDNA.

The expression levels of *SNCA*, *GBA1*, and *UGCG* were assessed via quantitative PCR (qPCR) using the LightCycler System (LightCycler^®^ SW 1.5, Roche Diagnostics GmbH, Mannheim, Germany). Duplicate 5 μL reactions were set up in 384-well plates using GoTaq qPCR Master Mix containing a dsDNA binding dye (Promega, Madison, WI, USA), 2 μL of 1:20 diluted cDNA, and 200 nM of the following gene-specific primers: 5′-ATGTTGGAGGAGCAGTGGTG-3′ and 5′-CTGTGGGGCTCCTTCTTCA-3′ for *SNCA*, 5′-ATGGAGCGGTGAATGGGAAG-3′ and 5′-GTGCTCAGCATAGGCATCCAG-3′ for *GBA1*, and 5′-GAATGGCCGTCTTCGGGTT-3′ and 5′-AGGTGTATCGGGTGTAGATGAT-3′ for *UGCG*. The cycling conditions for all three genes included an initial denaturation step at 95 °C for 2 min, followed by 50 cycles of amplification (3 s denaturation at 95 °C, 20 s annealing and amplification at 60 °C), and a cooling step at 40 °C for 30 s. A melting profile was incorporated to verify product specificity. The expression levels of reference genes beta-actin (*ACTB*), ornithine decarboxylase 1 (*ODC1*), and beta-2-microglobulin (*B2M*) were determined in a multiplex qPCR using double fluorescently labeled probes, as previously described [84]. Quantification cycles were determined using the “Abs Quant/2nd Derivative Max” analysis method provided by the LightCycler Software. The geometric mean of the duplicates was adjusted for separately determined gene-specific efficiencies. Each target gene expression was normalized to the geometric mean of the three reference genes. Finally, outliers lying beyond 2.5 standard deviations from the mean based on logarithmized expression values were removed (1 and 2 for *SNCA* for T1 and T2, respectively, 3 and 3 for *GBA*, 3 and 3 for *UGCG*).

### 4.5. Statistics

The data underwent analysis using IBM SPSS Statistics Version 29 for Windows (SPSS Inc., Chicago, IL, USA) and were visualized using GraphPad Prism 9.5.1 (733) (GraphPad Software Inc., San Diego, CA, USA). We utilized nonparametric methods as the gene expression values did not follow a normal distribution, as confirmed by the Kolmogorov–Smirnov test. Bivariate correlations were evaluated using Spearman’s method. Group differences for continuous variables were tested using the Mann–Whitney U test and for nominal variables using the χ^2^ test, while differences between inclusion and follow-up were assessed using the Wilcoxon signed-rank test for related samples. Continuous data were expressed as the median and interquartile ranges within tables, calculated using SPSS’s custom tables function. Subjects with missing data points were excluded from specific analyses. Three individuals were completely excluded for the lack of any gene expression data at T1. A significance level of *p* < 0.05 for two-sided tests was considered nominally statistically significant. To maintain transparency, *p* values were not corrected for multiple testing, and nominal *p* values are reported in tables and graphs. Initially, female and male subjects were analyzed together for primary hypotheses, followed by explorative sex-specific analysis due to well-established sex differences in depression [58].

## 5. Conclusions

In summary, our findings reveal a significantly elevated peripheral gene expression of *SNCA*, *GBA1*, and *UGCG* among patients currently experiencing depression which partially normalizes for *GBA1* and *UGCG* in the group of medicated patients and also reflects increased depression severity in subgroups. These findings further support the convergent mechanisms between depression and PD [91] involving sphingolipid pathways [92]. They hold promise for informing the development of diagnostic biomarkers and elucidating pathological mechanisms of depression, with the ultimate goal of advancing novel therapeutic approaches. Given their association with PD, these genes may also suggest specific overlapping pathomechanisms, prompting further investigation into potential biological interactions between these two conditions.

## Figures and Tables

**Table 1:** Study cohort description and group differences for unmedicated, medicated, and remitted patients and control subjects at study inclusion (T1) and follow-up (T2, 3 weeks later).

	Unmed. Patients		Medicated Patients		Remitted Patients		Healthy Controls			
	*n*	Median (IQR)	*n*	Median (IQR)	*n*	Median (IQR)	*n*	Median (IQR)	*p* (Sex)	*p* (Groups)
Age (years)	63	46 (33–53)	66	46 (33–54)	38	50 (46–58)	60	41 (32–54)	0.081	0.502
Education (years)	56	15 (13–18)	58	14 (13–16)	34	14 (13–16)	50	16 (13–18)	**0.008**	0.594
BMI (kg/m^2^)	63	25.1 (22.5–27.4)	66	28.5 (24.4–30.4)	38	25.7 (23.0–29.1)	60	24.5 (23.0–27.8)	**0.005**	0.190
HAM-D T1	63	21 (19–24)	66	23 (20–26)	38	2 (0–3)	60	0 (0–2)	0.743	**<0.001**
HAM-D T2	59	18 (14–20)	60	15 (10–22)					**0.048**	
MADRS T1	63	26 (23–28)	66	28 (24–34)	38	1 (0–3)	60	0 (0–2)	0.950	**<0.001**
MADRS T2	59	21 (18–25)	60	18 (13–26)					0.052	
BDI-II T1	63	28 (22–34)	66	29 (24–35)	38	3 (0–3)	60	2 (0–3)	0.528	**<0.001**
BDI-II T2	59	19 (15–25)	60	20 (13–31)					**0.009**	
STAI state T1	63	50 (40–56)	66	54 (43–63)	38	32 (26–36)	60	28 (26–31)	0.910	**<0.001**
STAI state T2	59	46 (37–52)	60	47 (42–57)					0.819	
STAI trait mean	63	60 (55–66)	66	58 (52–66)	38	34 (27–40)	60	28 (25–33)	0.624	**<0.001**
*SNCA* expression (AU) T1	63	17.7 (9.8–26.3)	66	21.2 (10.1–36.2)	38	13.8 (7.6–34.2)	59	15.6 (6.7–29.3)	0.666	**0.036**
*SNCA* expression (AU) T2	55	14.2 (8.2–25.5)	60	20.3 (9.9–31.3)					0.420	
*SNCA* expression rel. change	55	−0.016 (−0.450–0.656)	60	−0.044 (−0.370–0.556)					0.322	
*GBA1* expression (AU) T1	63	0.237 (0.136–0.474)	64	0.302 (0.177–0.458)	38	0.194 (0.072–0.395)	59	0.178 (0.081–0.429)	0.570	**0.014**
*GBA1* expression (AU) T2	56	0.208 (0.099–0.394)	57	0.245 (0.149–0.493)					0.255	
*GBA1* expression rel. change	56	−0.131 (−0.587–0.873)	56	−0.210 (−0.444–0.201)					0.435	
*UGCG* exp. (AU) T1	63	0.136 (0.070–0.200)	64	0.174 (0.105–0.291)	38	0.093 (0.048–0.173)	57	0.089 (0.047–0.215)	0.123	**<0.001**
*UGCG* exp. (AU) T2	57	0.122 (0.072–0.229)	57	0.147 (0.092–0.271)					0.147	
*UGCG* exp rel. change	57	0.173 (−0.577–1.018)	55	−0.227 (−0.499–0.178)					0.431	

The table shows frequencies (*n*) and median with interquartile range (IQR) at inclusion (T1) and follow-up (T2, at median 3 weeks later) and *p* values (nominal *p* < 0.05 in bold) from Mann–Whitney U tests comparing females with males (*p* (sex)) or patient groups (combined PU + PM) with controls (combined PR + HC). See Supplementary Table S1 for all group-wise comparisons. Sex-separated group sizes (females/males at T1 and T2, respectively)—PU unmedicated depressive patients (37/26; 34/25), PM medicated depressive patients (32/34; 28/32), PR patients with remitted major depressive disorder (28/10) and HC healthy controls (30/30). χ^2^ test for T1 sex distribution (PU + PM versus PR + HC) *p* = 0.392. Parameters—BMI body mass index, BDI-II Beck Depression Inventory-II, HAM-D Hamilton Depression Rating Scale, MADRS Montgomery–Åsberg Depression Rating Scale, STAI State–Trait Anxiety Inventory, peripheral gene expression for *SNCA* α-synuclein, *GBA1* β-glucocerebrosidase, *UGCG* UDP-glucose ceramide glucosyltransferase in arbitrary units (AU) normalized to reference genes.

**Table 2:** Correlation of *SNCA* expression with depression severity scores as assessed by self-rating (BDI-II) in unmedicated (PU) and medicated (PM) patients with major depressive disorder at inclusion, ρ, and *p* bold for *p* < 0.05.

*SNCA* Expression in PU + PM	HAM-D			MADRS		BDI-II	
	*n*	ρ	*p*	ρ	*p*	ρ	*p*
All	129	0.016	0.855	0.107	0.227	**0.190**	**0.031**
Female	69	0.150	0.219	0.197	0.105	**0.256**	**0.034**
Male	60	−0.130	0.323	−0.030	0.818	0.046	0.726

**Table 3:** Correlations of *SNCA*, *GBA1*, and *UGCG* expression with depression severity (assessed by HAM-D, MADRS, BDI-II) and trait anxiety (STAI) at inclusion in patients with remitted MDD, ρ, and *p* bold for *p* < 0.05.

	Remitted		HAM-D		MADRS		BDI-II		STAI Trait	
	Patients	*n*	ρ	*p*	ρ	*p*	ρ	*p*	ρ	*p*
*SNCA*	All	38	**0.363**	**0.025**	**0.350**	**0.031**	0.186	0.263	**0.325**	**0.047**
	Female	28	0.366	0.056	**0.387**	**0.042**	0.237	0.224	0.338	0.079
	Male	10	0.331	0.351	0.259	0.471	−0.032	0.931	0.273	0.446
*GBA1*	All	38	**0.336**	**0.039**	**0.404**	**0.012**	0.278	0.091	0.313	0.056
	Female	28	0.288	0.137	**0.390**	**0.040**	0.295	0.128	0.231	0.237
	Male	10	0.248	0.490	0.259	0.471	0.127	0.726	0.358	0.310
*UGCG*	All	38	**0.327**	**0.045**	**0.380**	**0.019**	0.262	0.111	**0.404**	**0.012**
	Female	28	0.344	0.073	**0.475**	**0.011**	0.308	0.111	**0.385**	**0.043**
	Male	10	0.133	0.713	0.032	0.929	−0.025	0.944	0.248	0.489

**Table 4:** Correlations of *SNCA*, *GBA1*, and *UGCG* expression with lactate dehydrogenase (LDH) and creatine kinase (CK) at inclusion in patients with remitted MDD and the combined group of healthy controls and remitted patients; rho and *p* bold for *p* < 0.05.

		*SNCA*			*GBA1*		*UGCG*	
		*n*	ρ	*p*	ρ	*p*	ρ	*p*
LDH	All	38	**−0.346**	**0.033**	**−0.421**	**0.008**	**−0.377**	**0.020**
Remitted	Female	28	−0.180	0.359	−0.283	0.144	−0.250	0.199
Patients	Male	10	**−0.869**	**0.001**	**−0.888**	**0.001**	**−0.699**	**0.024**
CK	All	97	−0.115	0.261	−0.183	0.073	−0.154	0.136
Healthy controls	Female	58	−0.000	0.999	−0.033	0.809	−0.033	0.806
and remitted patients	Male	39	**−0.353**	**0.027**	**−0.384**	**0.015**	**−0.379**	**0.019**

## Data Availability

Data are available upon request.

## Supplementary Materials

The following supporting information can be downloaded at: https://www.mdpi.com/article/10.3390/ijms25063219/s1.
