# Supplementary material for: Peripheral Upregulation of Parkinson’s Disease-Associated Genes Encoding α-Synuclein, β-Glucocerebrosidase, and Ceramide Glucosyltransferase in Major Depression
Source: Int J Mol Sci. 2024 Mar 12;25(6):3219. doi: 10.3390/ijms25063219 (PMC10970259; doi:10.3390/ijms25063219)
Supplement: Supplementary file 1 [file ijms-25-03219-s001.zip › ijms-2877829-supplementary.pdf]

International Journal of *Molecular Sciences*

MDPI

Article

Peripheral Upregulation of Parkinson's Disease-Associated Genes Encoding α-synuclein, β-glucocerebrosidase, and Ceramide Glucosyltransferase in Major Depression

Razvan-Marius Brazdis, Claudia von Zimmermann, Bernd Lenz, Johannes Kornhuber, Christiane Mühle

Supplementary Table S1: Group-wise comparisons (p values) for unmedicated, medicated and remitted patients and healthy control subjects at study inclusion (T1) and follow-up (T2, 3 weeks later, only for currently affected patients)

p values for pair-wise comparisons	PU-PM	PU-HC	PM-HC	PR-HC	PU-PR	PM-PR
Age	0.908	0.520	0.530	0.017	0.055	0.036
Education	0.083	0.937	0.066	0.220	0.220	0.689
BMI	**<0.001**	0.616	0.001	0.362	0.192	0.080
HAM-D T1	0.087	**<0.001**	**<0.001**	**0.017**	**<0.001**	**<0.001**
HAM-D T2	0.237					
MADRS T1	0.064	**<0.001**	**<0.001**	**0.022**	**<0.001**	**<0.001**
MADRS T2	0.171					
BDI-II T1	0.371	**<0.001**	**<0.001**	0.376	**<0.001**	**<0.001**
BDI-II T2	0.970					
STAI state T1	0.065	**<0.001**	**<0.001**	**0.020**	**<0.001**	**<0.001**
STAI state T2	0.237					
STAI trait average	0.332	**<0.001**	**<0.001**	**0.003**	**<0.001**	**<0.001**
SNCA expression T1	0.309	0.160	**0.038**	0.842	0.366	0.186
SNCA expression T2	0.157					
SNCA expression rel. change	0.840					
GBA1 expression T1	0.182	0.265	**0.043**	0.515	0.128	**0.017**
GBA1 expression T2	0.159					
GBA1 expression rel. change	0.830					
UGCG expression T1	**0.028**	0.055	**<0.001**	0.903	0.083	**<0.001**
UGCG expression T2	0.296					
UGCG expression rel. change	0.321					

p values (nominal $p < 0.05$ in bold) from Mann-Whitney-U tests comparing patient groups: PU unmedicated depressive patients, PM medicated depressive patients, PR patients with remitted major depressive disorder and HC healthy controls. Parameters—BMI body mass index, BDI-II Beck Depression Inventory-II, HAM-D Hamilton Depression Rating Scale, MADRS Montgomery–Åsberg Depression Rating Scale, STAI State-Trait Anxiety Inventory, peripheral gene expression for *SNCA* α-synuclein, *GBA1* β-glucocerebrosidase, *UGCG* UDP-glucose ceramide glucosyltransferase normalized to reference genes
